# Collectanea Medica: Consisting of Anecdotes, Facts, Extracts, Illustrations, &c.

**Published:** 1824-08

**Authors:** 


### COLLECTANEA MEDICA:

CONSISTING OF

ANECDOTES, FACTS, EXTRACTS, ILLUSTRATIONS, &c.

*Relating to the History or the Art of Medicine, and the Collateral Sciences.*

Floriferis, ut apes, in saltibus omnia libant,

Omnia nos, itidem, depascimur aurea dicta.

ART. I.—*Case of Chorea.* By JOHN CRAMPTON, M.D. &c. &c. &c. —(Transactions of the Association of Fellows and Licentiates of the King and Queen's College of Physicians in Ireland, Vol. IV.)

THE attention of anatomists and pathologists having been devoted, with considerable success, to most of the disorders with which the cerebral organs are engaged, our knowledge of them is thereby proportionally extended, and the mode of treating them very much improved. Materials for instruction on the subjects of apoplexy and palsy, so far as morbid anatomy can illustrate these diseases, have been amply collected. Even on epilepsy no small share of light has been thrown; but on chorea, which by most medical writers has been considered a disorder of the nervous system, anatomical research, I believe, affords no information.

Practical writers have, however, endeavoured to supply the deficiency, and experience has furnished us with many remedies, as well as with different successful modes of treating this disorder.

In the following case of Chorea, nitrate of silver was given freely, and, whilst the patient took it, she soon recovered her health. The case occurred in an adult, at a time of life when such a disease was little to be expected; but it is not the only instance of chorea to be met with at an advanced age, as Dr. Maton relates the history of a woma , æt. seventy, who was cured of that disorder by musk.*

CASE.—Anne M'Kelvey, æt. forty-two, unmarried, of a thin habit of body and pale complexion, was admitted into the Whitworth Hospital House of Industry, on 12th of February, 1821, labouring under a severe paroxysm of chorea; each hand and leg, sometimes one, sometimes all, were affected with a hurried convulsive motion, which appears to a spectator as if she had been beating time to a musical instrument. She speaks in a hurried manner, and expresses great uneasiness when the hand, or limb affected, is held, or attempted to be held: any restraint in this respect gives rise to convulsive motions in some other quarter. The present paroxysm has lasted five days. Bowels constipated; tongue looks unhealthy; pulse weak and slow; no catamenia the last two years.

Previous to September, 1819, had uninterrupted good health; at this time she became affected with hysteria, and was subject to attacks of it until about twelve months since: these consisted first in alternate fits of laughing and crying; afterwards a sensation of globus was experienced in her right side; but it was not until the last four months antecedent to her admission into the hospital, that these fits were exchanged for the motion in the extremities and the head already described. Since that period she has never been eight, seldom four, or even three, days without a return, which continues generally from three to five days, with little or no intermission. She has also, during the last four years, been frequently subject to a dull pain in the right temple: this appears to come on without any connexion in point of time with the convulsive affection. She finds all her symptoms are aggravated when her bowels are costive.

On her admission (12th of February), she was directed to have a fetid enema, after which the paroxysm subsided; also pills, with Rheum; Ext. hyosciami; Pil. hydrargyri; Extr. colocynth. comp. in nearly equal proportions, to be made into pills, of which two were to be taken every night. She was further ordered Argenti nitrat. gr. ¼, ter die.

15th.—No return of chorea. Argenti nitrat. gr. ss. ter die.

16th.—A severe paroxysm, removed by the enema.

19th.—A paroxysm of four hours, removed again by the enema; head-ache. Bol. ex calom. gr. iii.; Jalap, gr. viii.

22d.—Argenti nitrat. gr. i. ter die.

27th.—A severe paroxysm, removed immediately by an enema, with Olei terebinthinæ ʒii.; Tinct. opii ʒi. The enema was retained all night.

March 1st.—Argenti nit. gr. iss. ter die.

6th.—Argenti nit. gr. ii. bis die.

8th.—Baln. tepidum.

---

* *Med. Trans. Coll. Phys. Lond.* vol. v. p. 188.

9th.—Severe head-ache. Hirud. viii. tempori dextro. This relieved the pain.

12th.—Argenti nit. gr. ii. ter die.

16th.—Baln. tepidum.

20th.—Omit. argenti nitras. Rep. pil. ut 12 Feb. præscriptæ.

She was now discharged from the hospital, having been an entire month free from a return of the paroxysm, and remanded to the pauper department of the House of Industry.

May, 1822.—She has had no return of chorea; but, whenever there is any interruption to the regularity of the bowel-discharge, she is affected with severe head-ache, and occasionally with slight threatenings of hysteria.

The subject of the foregoing case, previous to her admission into the Whitworth Hospital, had been a resident in the pauper department of the House of Industry, where every attention had been paid to her health, and particularly to the state of her bowels. She took constantly, under the judicious direction of Dr. Orpen, laxative pills combined with fetids, besides other appropriate remedies, to combat a nervous affection of the character described. It is calculated she took above five hundred aperient pills, in varied forms, within the last twelve months; so that, if steady purgation, aided by a well-arranged diet and regular habits, could have subdued her disorder, many advantages for her relief were afforded. The disorder, however, scarcely remitted under any mode of treatment, until her removal to the hospital, and until the nitrate of silver was directed.

The circumstances under which she was placed in the Whitworth Hospital, it is but right to remark, were much more favourable to her recovery than when she was in the pauper department. In the hospital she was in a cheerful airy ward, containing only ten beds; whereas, in the repository for the poor, the apartment which she occupied had seventy beds, and its inmates, for the most part, remained either completely sedentary, or employed only in those kinds of work which require no exercise, and scarcely any muscular exertion.

The caustic nature of the remedy employed in this case, makes us justly careful in giving it inwardly; it has nevertheless been extensively employed in epilepsy and hysteria, as well as in chorea, in these countries, and not, as far as I can learn, with any unpleasant effects resulting from its use. I have known patients, who took it for epilepsy, complain in a few instances of griping pain, but not more than what we often observe from purgatives; and I have never observed such sensations continue for any time. Nor (although I have been particular in my inquiries,) have I heard patients complain of thirst or inward heat of stomach, symptoms which so often follow the employment of other acrid remedies. The change also in the colour of the skin, first described by Dr. Albers,* of Bremen, and afterwards by some of the British practitioners,† has not, as far as I can learn, been often observed in Ireland. My friend, Dr. Brooke, relates an instance where he gave

* *Med.-Chir. Trans.* vol. vii. p. 284.

† *Ibid.* vol. ix. p. 234.

the remedy in epilepsy with advantage, but where the blue colour of the skin ensued.

Sydenham may be considered as one of the first who described chorea, and who offered any pathology, or proposed any settled mode of treatment. He conceived it was a disease peculiar to the time of puberty; at this period, in both sexes, he states there is a considerable revolution, which induces plethora—in the male from the development of the sexual organs, and in the female from the impending menstrual effort. His mode of treatment was repeated bleedings, to diminish the revolutionary plethora, followed by frequent purging, dry cupping, and blisters to the affected limbs, and finally the cinchona bark and cold bathing.

In the case before us, the followers of Sydenham might say the period of the cessation of the catamenia is equally important with that of puberty in females; that, in this patient, the disappearance of the menstrual discharge was first followed by hysteria, and subsequently the nervous affection was exchanged for another, in many respects resembling it. Sydenham's practice might answer for full women, past forty, ceasing to menstruate, but it was not at all calculated for an emaciated female, as above described, at the period she came under treatment.

Some of the French practitioners,* adopting in part the pathology of Sydenham, apply leeches to the anus and pudenda; following this practice by giving remedies which they suppose act on the nerves, as the vegetable narcotics, bitters, and fetids, besides minerals, as zinc, iron, and copper. This practice is accompanied, as in most other cases, by giving mucilaginous demulcents and ptisans, with a constant use of lavements. In some instances camphor is given in injection, and electuary occasionally applied to the affected limbs, besides opiate frictions externally.

Since Dr. Hamilton, of Edinburgh, published his book on Purgatives, the profession has for the most part acted on his views. A steady purgative discipline has certainly, in many instances, succeeded; in some, however, it has been necessary to adopt other measures subsequently.

Dr. Powel† mentions several cases of chorea, treated by him in St. Bartholomew's Hospital with nitrate of silver, in which the doses were pushed to a much greater extent than in the case before us,—so far as even thirteen and fourteen grains in the day.

The arsenical solution has been employed with the same views. Mr. Thomas Martin‡ gave this mineral in a case of chorea, with success; and Mr. Salter,§ of Poole, prescribed it with advantage in a case which had resisted nitrate of silver, as well as most other remedies. Dr. George Gregory‖ also gave it at St. George's and St. James's Dispensary. These cases are related in the Medico-Chirurgical Transactions.

---

* *Dictionnaire des Sciences Medicales.*

† *Med. Trans. Coll. Phys. Lond.* vol. iv. p. 85.

‡ *Med.-Chir. Trans.* vol. iv. p. 45.

§ *Ibid.* vol. x. p. 218.

‖ *Ibid.* vol. xi. p. 299.

A case of chorea, occurring in a woman of seventy, is given by Dr. Maton,* in which the cure was effected by doses of musk continued for a considerable time.

Did chorea consist merely in distension or over-repletion of the vascular system in the brain, and that this state again took its rise from congestion in the liver and other viscera in the abdomen, with a general deficiency of secretion, Dr. Hamilton's purgative plan alone ought to prove more uniformly certain in affording relief. That there is something more than this in chorea, will readily be admitted by those who have witnessed the impression made by other modes of treatment, and especially by those medicines commonly called nervous, or antispasmodic, as in Dr. Maton's case. A peculiar, and often salutary, impression is made by under-doses of the narcotic vegetables, such even as in full doses are hurtful. The action of the mineral poisons on the cerebral organs, or nervous system, in chorea, has been illustrated by Dr. Powel's, Mr. Martin's, and Mr. Salter's cases, already referred to. This impression may be effected by the remedy diminishing an excessive animal sensibility, or by a direct abstraction of morbidly increased vital power; and the operation of nitrate of silver may possibly act by some influence analogous to this.

Chorea is not reckoned a fatal disease, (I believe we have no dissections on record of that disorder,) but it frequently gives a great deal of trouble, and undermines the constitution; so that those who have suffered from it readily fall victims to other diseases. Perhaps, as danger is not immediately in view, it does not sufficiently excite the apprehension of parents, and is not consigned sufficiently early to judicious medical treatment.

In confirmation of this, I may be allowed to state that, some years since, three children, of the same family, from eight to eleven years old, whose parents were consumptive, were placed under my care for chorea, which had long baffled domestic and other treatment. By removal to the country, by regular purgation, and by the employment of oxyd of zinc, aided by a suitable diet and exercise, the chorea was removed, and their general health seemed amended. The following winter they had hooping-cough; and, in a peripneumonic attack, which supervened during its progress, two of them were quickly carried off, although so little apprehension was entertained, that the relatives did not send for medical assistance until it was too late. Had suitable medical treatment been resorted to, in the early stages of chorea, with these patients, so that their constitutions should not have suffered by the long continuance of the nervous disorder, they might have resisted the attack of hooping-cough, with better prospects of recovery.

A very severe case of chorea, for which I was consulted, occurred in a boy of ten years old, whom I saw but twice. He had tried purgatives, vomits, tonics, and the other remedies usually ordered, besides other modes of relief suggested by different practitioners in the country. Head-ache was the prominent symptom. I directed leeches to the

---

* *Med. Trans. Coll. Phys. Lond.* vol. v. p. 188.

temples, neck, and along the spine, in succession, at the interval of a few days; shaving the head, using the tepid shower-bath, made gradually quite cold. These measures, with a proper attention to the state of the bowels, in the course of a few months restored him to health.

Although certain general principles should guide our practice, yet it would appear that no settled plan, no specific remedies, can apply generally to all cases of chorea we may meet with. Our measures must be varied according to the particular organ, or system, which seems to suffer most. At one time, our views must be directed to the vascular system in the head, also to the state of the liver and digestive organs; again, the condition of the nervous system chiefly claims attention. In other instances, a combined treatment must be adopted, using at the same time both evacuants and nervous medicines.

The influence of change of air, of cold-bathing, of cheerful society, and of exercise, are not to be overlooked: the treatment, in fact, ought to embrace, not only an accurate regulation of the functions of the digestive organs, but an attention to the state of the vascular system in the head, in addition to that of the cerebral or nervous system. But other considerations should likewise claim our notice: the condition of the spinal column, and the system of nerves which emanate from it; also the state of some particular nerves, which may have sustained a degree of local injury, in which instance a judicious local treatment must be combined with general remedies.

But it would extend the limits of this paper too far, were I to enter more fully into the subject of chorea; indeed, it has already exceeded the bounds which were intended: I shall therefore conclude, requesting those who may meet with cases of this malady which end unfavourably, or should its subjects be carried off by other disorders unexpectedly supervening, that they may not fail to make themselves acquainted with the morbid appearances, and communicate the result to the medical world. This will be the first and most necessary step to acquire a more thorough acquaintance with chorea.

